# Effects of Training Nursing Students on Female Genital Mutilation: A Quasi‐Experimental Study With Pre‐ and Postintervention Assessments

**DOI:** 10.1155/nrp/9450657

**Published:** 2026-10-11

**Authors:** Souad Ayad-Ouchen, José Antonio Jiménez-Barbero, Yusuly Mitkany Martínez-Jurado, María del Mar Pastor-Bravo

**Affiliations:** ^1^ Faculty of Nursing University of Murcia Buenavista Avenue, 32, El Palmar Murcia 30120 Spain um.es; ^2^ Faculty of Nursing University of Murcia Murcia Institute for Biosanitary Research Pascual Parrilla (IMIB) Buenavista Avenue, 32, El Palmar Murcia 30120 Spain um.es; ^3^ Faculty of Nursing and Obstetrics Autonomous University of the State of Mexico Paseo Tollocan Avenue & Jesús Carranza Street, Colón and Col Ciprés Neighborhoods Toluca de Lerdo 50120 Mexico uaemex.mx

**Keywords:** attitudes, female genital mutilation, health knowledge, nursing students, problem-based learning

## Abstract

**Aim:**

This study aimed to examine the association between an active learning‐based educational intervention and changes in nursing students’ knowledge, attitudes, and clinical management regarding female genital mutilation/cutting (FGM/C).

**Methods:**

A quasi‐experimental pre–post study without a control group was conducted during the 2024–2025 academic year at a public university in southeastern Spain. Sixty‐nine third‐year nursing students completed a validated questionnaire that assessed their general knowledge, attitudes, and clinical management of FGM/C before and within 7 days after the intervention. The program included a theoretical session and a practical case‐based learning session. Pre–post differences were analyzed using the Wilcoxon signed‐rank test, with effect sizes calculated as *r*.

**Results:**

General knowledge significantly improved after the intervention (*p* < 0.001, *r* = 0.42). A small statistically significant change was observed in attitudes (*p* = 0.020, *r* = 0.28); however, this finding should be interpreted cautiously given the low internal consistency of the attitude subscale. No significant differences were found in clinical management knowledge (*p* = 0.300, *r* = 0.12).

**Conclusion:**

The active learning‐based training was associated primarily with short‐term improvements in nursing students’ general knowledge of FGM/C, while changes in attitudes were limited and no improvements were observed in clinical management knowledge. Although changes were observed in the attitude domain, these findings should be interpreted cautiously due to the low internal consistency of the corresponding subscale. Further controlled longitudinal studies using reliable outcome measures are needed to confirm these findings.

## 1. Background

Female genital mutilation/cutting (FGM/C) refers to practices that involve the partial or total removal of the female external genitalia, as well as other injuries to the female genital organs for nonmedical reasons. The World Health Organization (WHO) categorizes FGM/C into four types: (a) Type I or clitoridectomy (partial or total removal of the clitoral glans), (b) Type II or excision (partial or total removal of the clitoral glans and labia minora), (c) Type III or infibulation (narrowing of the vaginal opening), and Type IV (this includes all other procedures that cause injury to the female genitalia without medical justification) [1]. FGM/C is most commonly performed on girls between childhood and adolescence; the age range typically spans from birth to 15 years, although this can vary by country, community, and cultural group [2, 3]. The challenge of eradicating FGM/C is compounded by the fact that it is deeply rooted in social norms and perpetuated by cultural beliefs that emphasize collective identity. Various sociocultural motivations, such as the belief that FGM/C ensures female chastity and fidelity in polygynous societies, the perceived social and marital value of girls, and its interpretation as a rite of passage, help maintain this practice. The sociocultural dynamics surrounding gender inequality are closely connected to FGM/C, which is often used to control women’s sexuality and reinforce traditional gender roles [4, 5].

Globally, it is estimated that over 230 million women and girls have undergone FGM/C, with the highest prevalence found in Africa, Asia, and the Middle East [1]. In Europe, approximately 600,000 women have experienced FGM/C, and an estimated 180,000 girls are at risk of it [6]. In Spain specifically, there are about 80,282 women from countries where FGM/C is prevalent, with significant populations originating from Senegal, Nigeria, and Mali [7].

The consequences of FGM/C include a range of complications whose severity depends not only on the type of FGM/C practiced but also on factors related to the overall health of girls and women. These factors may include malnutrition, immunodeficiencies, and other diseases, as well as the conditions under which the procedure is performed [8–10]. FGM can lead to physical complications that may occur in the short term, such as intense pain, bleeding, and infections, including severe hemorrhage, septic shock, and, in extreme cases, death [1, 11]. Long‐term complications include genital scarring, menstrual disorders, and infertility. Additionally, psychological complications can arise, including anxiety, low self‐esteem, and posttraumatic stress disorder. These long‐term effects can persist throughout a woman’s life and significantly impact her quality of life, social relationships, and mental health [1, 12]. FGM/C can also result in problems related to sexual function, such as dyspareunia (pain during intercourse), poor sexual satisfaction, and a lack of sexual desire. Obstetric complications can occur as well, such as perineal tears, the need for cesarean deliveries, and postpartum hemorrhage, among others [11, 12].

FGM/C violates the rights to health and bodily integrity for girls and women. It also breaches the Convention on the Rights of the Child, as it is a traditional practice that harms children’s health [5]. Beyond its health impact, FGM/C also represents a barrier to gender equality, as it can restrict girls’ educational trajectories, limit their autonomy and participation in society, and perpetuate cycles of discrimination and inequality [4, 5]. To combat this issue, Sustainable Development Goal 5 aims to achieve gender equality and empower all women and girls, with target 5.3 specifically focusing on the elimination of harmful practices like FGM/C by 2030 [4]. The European legal framework addresses FGM/C through the Istanbul Convention, a binding instrument designed to prevent and combat violence against women, including female genital mutilation, as outlined in Article 38 [13]. In Spain, FGM/C is classified as a crime of severe injury under Article 149.2 of the Penal Code. This provision imposes penalties ranging from 6 to 12 years in prison for individuals who perform any form of genital mutilation. Additionally, if the victim is a minor or incapacitated, the court may impose a special disqualification from exercising parental authority, guardianship, custody, foster care, or curatorship for a period of 4–10 years, depending on what is deemed appropriate for the victim’s protection [14].

A 2021 study conducted in Tenerife found that only 19% of healthcare professionals surveyed had received specific training on FGM/C. Those who had received training demonstrated a greater ability to identify cases in their clinical practice [15]. Similarly, another study conducted in Castilla‐La Mancha that same year found that only 14% of professionals reported receiving training in this area [16]. These findings are particularly concerning given that many of these professionals serve at‐risk populations. This suggests that numerous cases may go undetected, and many affected women and girls may not receive the adequate care they need.

Women and girls who have undergone FGM/C represent a particularly vulnerable group that requires comprehensive healthcare tailored to their specific circumstances. In this context, the role of healthcare professionals is crucial, not only for addressing the consequences of FGM/C but also for preventing future cases [17]. Health professionals need to understand the arguments supporting the practice, as well as how to clarify and challenge these justifications. They should help families change their attitudes and raise awareness of their rights. Additionally, it is essential to explain and address the health consequences from a cultural perspective, while avoiding behaviors that may revictimize women and girls who have undergone this practice [18, 19].

A lack of training in cultural competencies, particularly regarding FGM/C, can hinder healthcare professionals’ understanding of communities in which FGM/C has sociocultural significance and create barriers in the care relationship. This is especially true in contexts where family decisions play a significant role. Such gaps in training can lead to increased frustration among professionals and diminish their ability to respond effectively [20, 21]. Additionally, the absence of specific training in FGM/C may result in the condition often going undetected and untreated in clinical settings [9].

Thus, this study aimed to assess the effects of a training program on FGM/C, utilizing active learning methodologies among third‐year nursing students.

### 1.1. Research Questions: This Study Sought to Explore the Following Research Questions

• Does the active learning training program improve nursing students’ general knowledge about female genital mutilation?
• Does the training program change nursing students’ attitudes toward female genital mutilation?
• Does the training program enhance nursing students’ clinical knowledge regarding the healthcare management of women affected by female genital mutilation?
• Are there significant differences between pre‐ and postintervention scores in general knowledge, attitudes, and clinical decision‐making responses?

## 2. Methods

### 2.1. Design

This study utilized a quasi‐experimental pretest and posttest design without a control group to assess changes associated with an educational program on FGM/C in nursing students. Given the absence of a control group, the findings should be interpreted as pre–post associations rather than evidence of causal effects attributable to the intervention.

### 2.2. Study Setting and Sampling

The subject “Nursing and Women’s Health” is a component of the Nursing Degree curriculum that focuses on providing nursing care to women throughout their life cycle, emphasizing the period from adolescence to old age. This course is part of the Nursing Bachelor’s Degree at a public university located in southeastern Spain, within the European Higher Education Area framework. As part of this subject, a study was conducted with third‐year nursing students at a public university in southeastern Spain during the 2024/2025 academic year. Participant recruitment and data collection were carried out between October 2024 and January 2025. A nonprobabilistic voluntary response sampling strategy was used. Third‐year nursing students enrolled in the course were invited to participate through the virtual classroom platform. The final study population consisted of students who voluntarily completed both the educational intervention and the study assessments according to the inclusion criteria. No formal sample size calculation or statistical power analysis was performed, as the study followed a quasi‐experimental educational design based on the total number of students available in the course during the 2024/2025 academic year.

### 2.3. Inclusion and Exclusion Criteria

The inclusion criteria for the study were as follows: participants had to be enrolled in the course during this academic year. They also had to have participated in the training program that utilized active learning methodologies regarding FGM/C. Students who did not attend both sessions of the training program, titled “Approaching FGM with Active Learning Methodologies,” were excluded, as were those who did not complete both the pretest and posttest questionnaires. The research team communicated the study’s objectives to the students who met the established inclusion criteria. Participation in the study was voluntary. All eligible students were invited to participate through the virtual classroom platform, and no incentives were provided.

### 2.4. Intervention

#### 2.4.1. FGM/C Education Program

The FGM/C educational program was designed using active learning methodologies as part of a systematic approach. This process included assessing students’ knowledge of FGM/C, their attitudes toward the practice, and their understanding of the health implications associated with it. To ensure the program was evidence‐based, the content was developed through a comprehensive literature review of existing studies related to the practice.

The program was divided into two sessions: (a) a theoretical session in which students learned about FGM/C, including its definition, the reasons it is performed, the health consequences associated with the practice, human rights issues, and current legislation regarding FGM/C, and |(b) a practical session for small groups of students, focusing on the “Protocol for the Prevention and Health Action regarding Female Genital Mutilation in the Region of Murcia.” This session involved resolving and discussing practical cases related to this traditional practice, all while ensuring the anonymity of the participating students.

#### 2.4.2. Procedure

##### 2.4.2.1. Preintervention Assessment

Student recruitment was conducted through an announcement in the Virtual Classroom. Third‐year nursing students enrolled in the “Nursing and Women’s Health” course were invited to participate anonymously in completing a previously validated questionnaire [22]. Participation in this questionnaire was optional. Additionally, they were invited to attend a theoretical class on FGM/C, during which the purpose of the study was explained to them.

##### 2.4.2.2. FGM/C Education Program for Nursing Students

The FGM/C training program was conducted during the 2024/2025 academic year and consisted of two 2‐hour sessions per course. Students were divided into three groups for the practical sessions. The program took place in a single classroom within a university’s Nursing Bachelor’s Degree program, with researchers facilitating all sessions.

##### 2.4.2.3. Posttest

The posttest involved participants recompleting the self‐administered questionnaire anonymously and voluntarily through the virtual classroom, within 7 days of finishing the program. A detailed description of the intervention, including learning objectives, session structure, teaching materials, case vignettes, and fidelity procedures, is provided in Supporting Information S1 to ensure reproducibility.

### 2.5. Measures

#### 2.5.1. Instruments

A previously validated questionnaire was utilized to assess the training program that employed active learning methodologies related to FGM/C [22]. The overall questionnaire showed a Cronbach’s alpha of 0.75 in the study sample. However, as this is a multidimensional instrument, reliability must be interpreted separately for each domain rather than as a single overall estimate. It comprises three dimensions:

a. The general knowledge dimension (Items 1, 2, 3, 4, 7, 8, 9, and 10) evaluates knowledge about FGM/C, including its typology, identification, causes, consequences, contributing factors, legislation, and protocols. This dimension demonstrated adequate reliability, with a Cronbach’s alpha of 0.80. The item related to previous exposure to training on FGM/C was excluded from this dimension and analyzed separately as a descriptive variable.
b. The attitudes dimension (Items 5 and 6) gauges students’ attitudes toward the practice of FGM/C and their willingness to participate in it. This dimension exhibited low reliability, with a Cronbach’s alpha of 0.40.
c. The clinical knowledge dimension, which focuses on the healthcare approach to FGM/C (Items 11, 12, 13, and 14), assesses students’ responses to various practical cases involving FGM/C. This dimension showed low reliability, with a Cronbach’s alpha of 0.33.

Item 15, *“Have you ever attended any course or training session on Female Genital Mutilation/Cutting?”* was considered a standalone descriptive item evaluating prior exposure to FGM/C training and was excluded from all dimensional scores and from the total questionnaire score.

Questionnaire responses were initially collected using the original response structure of the validated instrument. Subsequently, selected items were recoded during statistical processing in SPSS to improve score‐direction consistency across dimensions. For most knowledge items, higher scores reflected greater knowledge. For attitude items, interpretation depended on the direction of coding; in this study, lower scores indicated more appropriate attitudes toward FGM/C (i.e., greater rejection of the practice and its medicalization). Clinical management items (P11–P14) retained their original scoring structure based on the number and combination of management actions selected. To enhance transparency and reproducibility, Supporting Information S2 provides a detailed description of each questionnaire item, including domain allocation, response options, analytical coding procedures, and score direction. Because the questionnaire consisted primarily of categorical and ordinal variables that were numerically recoded for analytical purposes, item interpretation was based on coding direction rather than on uniform numerical score ranges across all items.

Finally, an open‐ended question was posed to identify which aspects a training program should emphasize to be beneficial in their professional practice.

### 2.6. Participant Flow

Participant flow was reported in accordance with the TREND statement for nonrandomized intervention studies. Students enrolled in the course were invited to participate through the virtual classroom platform. Sixty‐nine students voluntarily completed both the pretest and posttest assessments and attended the full educational intervention. All participants provided complete questionnaire data for both assessments. The flow of participants through the different stages of the study is shown in Figure 1. All analyses were conducted using the full sample of 69 participants with complete paired data. No observations were excluded due to missing responses, and no imputation methods were applied. Given the absence of a control group and the use of complete‐case analysis, results are interpreted as pre–post changes in the observed sample rather than causal effects attributable exclusively to the intervention.

**FIGURE 1:**
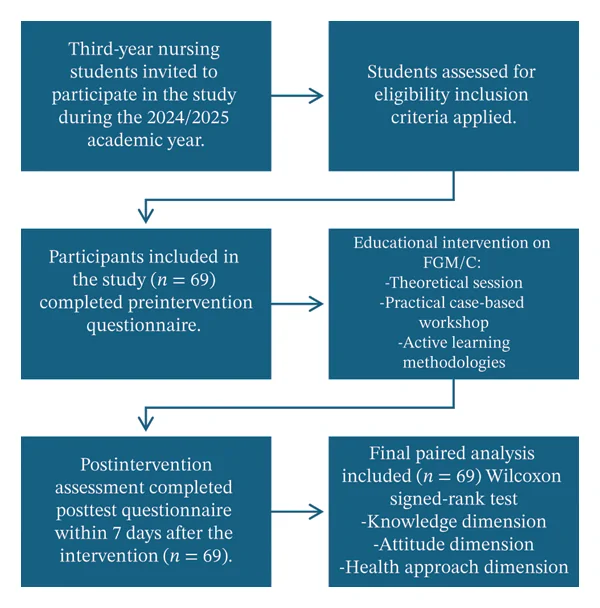
Note. Flow of participants through the educational intervention and outcome assessment. All 69 participants completed both the pre‐ and postintervention assessments and provided complete paired questionnaire data. All participants were included in the quantitative analyses.

### 2.7. Data Analysis

Data analysis was conducted using the Statistical Package for the Social Sciences (SPSS) Version 27. A *p* value of less than 0.05 was considered statistically significant. The questionnaire consisted of categorical variables that were coded numerically for quantitative analysis. The Kolmogorov–Smirnov test was used to assess data distribution, which indicated a nonnormal distribution. All participants completed both the pre‐ and postintervention assessments and provided complete questionnaire data. All paired analyses were conducted using the full sample of 69 participants, and no observations were excluded due to missing data. Therefore, the Wilcoxon signed‐rank test was applied to compare pre‐ and postintervention scores for paired observations within the same participants. Effect sizes (*r*) were calculated as *r = Z*/√*N*, where *Z* represents the standardized test statistic and N is the number of paired observations included in each analysis (*N* = 69). Effect sizes were interpreted according to conventional thresholds (0.10 = small, 0.30 = moderate, 0.50 = large). Confidence intervals were not calculated for the Wilcoxon‐derived effect sizes because these estimates are not routinely provided within the standard nonparametric framework used in this study. Therefore, effect magnitude was summarized using the *r* effect size metric. Additionally, responses to the open‐ended question were analyzed qualitatively. Responses to the open‐ended question were analyzed using a descriptive content analysis approach with an inductive coding process. Two researchers independently reviewed the responses, generated initial codes, and grouped them into categories. Any discrepancies in coding were resolved through discussion and consensus. No a priori sample size calculation was conducted due to the pragmatic and educational nature of the study, which included the entire accessible population of eligible students. Item‐level analyses were considered exploratory in nature; therefore, no correction for multiple comparisons was applied. Results from these analyses should be interpreted with caution due to the increased risk of type I error.

### 2.8. Ethical Considerations

This study was approved by the Research Ethics Committee of the University of Murcia (approval number: M10/2025/338MR1).

Participation was entirely voluntary. Before accessing the online questionnaire, all participants were required to provide electronic informed consent by selecting an acceptance checkbox after reading the study information sheet. Students were explicitly informed that participation or nonparticipation would have no academic consequences and would not affect their course of assessment, grades, or academic standing in any way.

Given that some members of the research team also held teaching and assessment responsibilities within the course, specific measures were implemented to minimize potential power imbalances. Participation was managed through the virtual classroom platform, questionnaire responses were collected anonymously, and individual responses were not accessible to teaching staff during the academic assessment process. Data analysis was conducted only after completion of course‐related evaluation activities.

Pre‐ and postintervention questionnaires were matched using self‐generated anonymous identification codes created by participants, ensuring paired analysis without direct personal identification.

Due to the sensitive nature of the topic, students were informed in advance about the content of the educational sessions. Following the intervention, a debriefing session was offered to address questions, clarify concepts, and provide an opportunity for discussion. Participants were informed of their right to withdraw from the study at any time without penalty. Participation or nonparticipation had no influence on academic assessment, course grades, or relationships with teaching staff.

Data confidentiality and processing were ensured in accordance with Regulation (EU) 2016/679 (General Data Protection Regulation) and Organic Law 3/2018 of December 5 on the Protection of Personal Data and Guarantee of Digital Rights.

The study was registered on the Open Science Framework (OSF): https://osf.io/agj5q.

## 3. Results

### 3.1. Sample Characteristics of Participants

All quantitative analyses were performed using complete paired data from the 69 participants who completed both the pre‐ and postintervention assessments. The mean age of participants was 24.48 years (see Table 1). No participants were excluded from the analyses because of missing data.

**TABLE 1:** Sociodemographic characteristics of the study sample.

Variables	Study group (*n* = 69)	
Age (mean and mode)	24, 48	20
Sex		
Female	52	75.4%
Male	16	23.2%
Nonbinary	1	1.4%
Academic course		
2024/2025	69	100%
Years of experience in a health‐related sector		
No experience	28	40.6%
< 1 year	19	27.5%
1–10 years	16	23.2%
10–20 years	4	5.8%
20–30 years	2	2.9%

### 3.2. Normality Testing and Distribution of Study Variables

In the normality test, a significance level of less than 0.001 was found. As a result, we rejected the null hypothesis and accepted the alternative hypothesis, which states that the data do not follow a normal distribution.

### 3.3. Pre–Post Differences in General Knowledge, Attitudes, and Clinical Knowledge on FGM/C

Wilcoxon signed‐rank tests (Table 2) revealed a statistically significant increase in knowledge about FGM (*Z* = −3.511, *p* < 0.001, *r* = 0.42), indicating a moderate effect size, with a higher number of positive ranks (*n* = 46) than negative ranks (*n* = 14), indicating a predominance of positive changes in participants’ knowledge following the intervention. A statistically significant change was also observed in attitudes toward FGM (*Z* = −2.324, *p* = 0.020, *r* = 0.28), corresponding to a small to moderate effect. In this case, the predominance of negative ranks (*n* = 18 vs. *n* = 11) reflects a decrease in scores, which, given the coding of the variable, suggests a shift toward more critical responses to FGM/C‐related scenarios after the intervention. However, this result should be interpreted with caution due to the low internal consistency of the attitude subscale (*α* = 0.40), which may limit the robustness of this construct‐level interpretation.

**TABLE 2:** Analysis of pre‐ and posttraining dimensions.

Dimensions	*N* paired observations	Premean (SD)	Postmean (SD)	Negative ranks (*n*)	Positive ranks (*n*)	*Z*	*p*	Effect size (*r*)
Knowledge about FGM (Items 1, 2, 3, 4, 7, 8, 9, and 10)	69	13.45 (2.67)	15.04 (1.77)	14	46	−3.511	< 0.001	0.42
Attitude toward FGM (Items 5 and 6)	69	0.62 (1.16)	0.25 (0.58)	18	11	−2.324	0.020	0.28
Knowledge regarding the health approach to FGM (Items 11, 12, 13, and 14)	69	6.64 (1.95)	6.25 (2.05)	31	22	−1.037	0.300	0.12

*Note:* Effect sizes are reported as *r* values derived from the standardized Wilcoxon signed‐rank test statistic (*r* = *Z*/√*N*). Values of 0.10, 0.30, and 0.50 indicate small, moderate, and large effects, respectively.

In contrast, no statistically significant differences were found in knowledge regarding the health approach to FGM (*Z* = −1.037, *p* = 0.300, *r* = 0.12). The distribution of ranks (31 negative vs. 22 positive) suggests variability in responses without a consistent direction of change, indicating that no measurable changes were observed in this dimension. This result should also be interpreted cautiously given the low internal consistency of the clinical knowledge subscale (*α* = 0.33), which may have limited its capacity to detect meaningful change.

### 3.4. Item‐Level Changes in Knowledge and Professional Response to FGM/C

Item‐level analysis revealed statistically significant differences in Items 1, 2, 6–10, and 13–15 (*p* < 0.05), with effect sizes ranging from small to very large (*r* = 0.30–0.95). The largest effects were observed for Items 7, 9, and 15. In contrast, no significant changes were found for Items 3, 4, 5, 11, and 12 (*p* > 0.05). Overall, the findings indicate heterogeneous patterns of change across individual questionnaire items, with statistically significant differences observed in some areas but not others (Table 3). Given the exploratory nature of the item‐level analyses, statistically significant findings should be interpreted cautiously because multiple comparisons may increase the likelihood of type I error. These findings should, therefore, be considered hypothesis‐generating rather than confirmatory.

**TABLE 3:** Analysis of pre‐ and posttraining dimensions by items.

Item	*N* paired observations	Premean (SD)	Postmean (SD)	Negative ranks (*n*)	Positive ranks (*n*)	*Z*	*p*	*r*
1	69	0.88 (0.37)	1.00 (0.00)	1	9	−2.530	0.011	0.30
2	69	3.25 (1.12)	3.93 (0.43)	2	23	−4.054	< 0.001	0.49
3	69	1.19 (0.81)	1.01 (0.21)	28	17	−1.732	0.083	0.21
4	69	3.96 (1.37)	3.49 (1.95)	25	18	−1.555	0.120	0.19
5	69	0.20 (0.58)	0.19 (0.43)	8	11	−0.357	0.721	0.04
6	69	0.42 (0.76)	0.06 (0.29)	18	3	−3.229	0.001	0.39
7	69	0.09 (0.41)	0.65 (0.51)	3	41	−5.689	< 0.001	0.68
8	69	1.74 (0.44)	1.96 (0.21)	2	17	−3.441	0.001	0.41
9	69	1.59 (0.63)	1.03 (0.34)	45	6	−5.158	< 0.001	0.62
10	69	0.68 (0.92)	1.00 (0.00)	21	43	−2.750	0.006	0.33
11	69	3.35 (1.28)	3.28 (1.39)	18	19	−0.552	0.581	0.07
12	69	0.57 (0.78)	0.54 (0.83)	22	20	−0.148	0.882	0.02
13	69	1.01 (0.87)	0.51 (0.83)	31	9	−3.481	0.001	0.42
14	69	1.71 (0.46)	1.93 (0.26)	5	20	−3.000	0.003	0.36
15	69	0.07 (0.26)	0.97 (0.17)	0	62	−7.874	< 0.001	0.95

*Note: N* = 69. Negative ranks indicate post < pre; positive ranks indicate post > pre. Effect size *r* = *Z*/√*N*. Lower scores in attitude indicate more appropriate positioning (greater rejection of female genital mutilation).

### 3.5. Students’ Perceived Educational Needs Regarding FGM/C Training

A total of 62.3% of students responded to the open‐ended question regarding the educational needs for training on FGM/C for professional practice. Responses are summarized in Table 4.

**TABLE 4:** What focus should a training action on FGM/C have to be useful in professional practice.

Answer	Frequency (pretraining)	Frequency (posttraining)
Knowledge of FGM	14	0
Education (survivors‐parents)	8	3
Action in a case of FGM	8	5
Raising awareness about consequences	6	1
Prevention	4	3
Survivor approach	3	2
Communication	2	0

Content analysis using an inductive descriptive approach identified several key areas for training improvement, including knowledge of FGM/C, educational interventions targeting families and women affected by FGM/C, clinical action in cases of FGM/C, awareness of consequences, prevention strategies, survivor‐centered care, and communication skills.

Participants particularly emphasized the need for more practical and applied training:

“We need more practical cases to know how to act in real situations.”

In addition, students highlighted the importance of communication and respectful care:

“Knowing how to talk to affected women without causing harm or judgment is essential.”

These findings reinforce the need to strengthen practical, case‐based components within FGM/C training programs.

### 3.6. Intervention Fidelity and Implementation

The intervention was delivered as planned without modifications during implementation. All sessions were conducted by the research team following a standardized protocol. Both theoretical and practical sessions were delivered in full according to the study design. Attendance records confirmed full participation in both intervention sessions among enrolled students. No deviations from the planned educational content were identified (See Supporting Information S1).

### 3.7. Adverse or Unintended Effects

No adverse effects or unintended negative consequences were reported by participants during or after the educational intervention.

## 4. Discussion

FGM/C remains a significant social and health issue, making it a priority topic in the training of health professionals. Numerous studies have highlighted a widespread deficiency in specific training regarding this practice among nursing students and other healthcare professionals [16, 23–25].

In the context of Spain, it has been documented that only 10.7% of professionals can accurately identify the types of FGM/C, and fewer than 9% are aware of an existing action protocol [16]. Despite possessing basic theoretical knowledge, many professionals do not feel equipped to intervene in actual cases, primarily due to a lack of practical training and unfamiliarity with standardized procedures [20, 26]. However, previous studies have suggested that educational interventions incorporating active methodologies such as workshops, clinical case analysis, simulations, or virtual dramatizations significantly enhance both knowledge and the confidence of professionals [27, 28]. Therefore, training for nursing students and various health professionals is essential, not only at the university level but also through ongoing education. This ensures they acquire the necessary knowledge to provide quality care to girls and women who have experienced genital mutilation [20, 24, 29]. Furthermore, these findings emphasize the importance of establishing periodic training programs for both students and practicing professionals. These programs should comprehensively address the clinical, ethical, legal, and cultural aspects of FGM/C, with the goal of providing culturally competent healthcare that focuses on human rights.

Knowledge of the legal framework and institutional protocols has significantly improved following the intervention. Spanish legislation recognizes FGM/C as a form of gender‐based violence and provides protection mechanisms for minors at risk. However, this legal framework has not yet been fully integrated into daily clinical practice, which hinders healthcare personnel’s ability to detect and respond appropriately [8]. At the national level, the Ministry of Health has approved the Common Health Action Protocol for Female Genital Mutilation to prevent, detect, and address this type of violence [30]. At the regional level, the Region of Murcia has established a Protocol for the Prevention and Health Action Against FGM/C. This protocol emphasizes the need for expanded training at various levels: in health degree programs, specialized health training, continuing education for professionals within the Murcian Health Service (SMS), and awareness‐raising initiatives among nonhealth organizations and the general public [31]. Overall, the findings of this research support the recommendations outlined in these protocols and underscore the need for effective implementation across all levels of the health system.

Significant changes were observed in areas related to the ability to act professionally in real‐life clinical settings, such as providing care to pregnant women who have undergone FGM/C and intervening with minors at risk. These results, based on questionnaire responses, suggest shifts in how students reported they would approach certain clinical scenarios involving FGM/C. The literature has shown that even with training, professionals may hesitate or resist intervention due to a lack of clear protocols and fears of legal or ethical repercussions [32]. The observed changes in these item‐level responses suggest that the intervention may have contributed to increased awareness and preparedness among participants; however, these findings should be interpreted cautiously given the absence of a control group and the low internal consistency of the clinical knowledge subscale. These questionnaire‐based findings should not be interpreted as evidence of actual clinical competence or professional performance and require confirmation through controlled studies using objective measures.

The growing number of students reporting prior training (such as courses, talks, or workshops) on FGM/C underscores both the potential educational value of these initiatives and the necessity of systematically integrating this topic into training programs. Recent reviews indicate that well‐designed educational interventions, particularly those utilizing active and participatory methodologies, can significantly enhance knowledge, attitudes, and professional self‐efficacy [20]. Therefore, it is recommended that FGM/C content be included as a mandatory and integral part of the university Health Sciences curricula. This should prioritize teaching methods that actively engage students and promote critical reflection on harmful cultural practices. These findings should, however, be interpreted cautiously, as statistically significant changes were primarily observed in the general knowledge dimension, while interpretation of the attitude and clinical management domains is constrained by the low reliability of these subscales. The observed findings, therefore, reflect changes in questionnaire‐measured responses and should not be interpreted as evidence of improved professional competence or clinical preparedness. Additional or more sustained educational strategies, together with objective assessments, may be required to determine whether training translates into measurable changes in professional performance.

The results regarding nursing students’ attitudes toward FGM/C show a partially positive change after their training. Specifically, in the question, “If FGM/C were performed under medical supervision, would you participate as a professional?,” a significant shift was observed. This indicates that students expressed lower willingness to participate in the medical practice of FGM/C, even when it is proposed to be conducted under sanitary conditions. This finding may reflect increased critical awareness regarding the medicalization of FGM/C following the educational intervention, leading to a more critical view of the “medicalization” of FGM/C. On the other hand, no significant changes were noted in response to the question, “Do you believe the practice of FGM/C should be respected as an option for geographical, cultural, religious, or other reasons?” This result shows that some deeply held cultural beliefs persist despite the training. This aligns with findings from a study in Guinea, where most healthcare providers opposed FGM/C and its medicalization. However, a considerable number still believed that cultural practices could justify it in specific contexts [33]. Similarly, a study in Somalia revealed that while healthcare providers recognized the harms associated with FGM/C, there remained a community belief that it might be acceptable if performed in safer conditions, highlighting the tension between cultural norms and public health principles [34]. These findings may support the inclusion of human rights, ethics, and intercultural reflection components within FGM/C educational programs. This approach will enable future professionals to act with cultural sensitivity while firmly opposing practices that violate the integrity of girls and women.

The present findings suggest that active learning methodologies may represent a useful educational approach for introducing FGM/C‐related content in nursing education. The intervention appeared to support improvements in theoretical knowledge of FGM/C. Changes were also observed in some attitude‐related outcomes; however, these findings should be interpreted cautiously given the low internal consistency of the attitude subscale. No significant improvement was observed in the clinical management knowledge domain.

It is essential to recognize that primary care healthcare professionals are often the first point of contact for girls or women who have undergone FGM/C or are at risk of it. To effectively address this issue, these professionals must receive proper training. Therefore, it is recommended that nursing students, both at the undergraduate and postgraduate levels, receive training that incorporates active learning methodologies focused on FGM/C. Research has shown that care provided by healthcare professionals who are knowledgeable about this issue significantly improves the support offered to survivors [35, 36]. In addition, implementing educational strategies targeted at communities can be an effective approach to reducing the prevalence of FGM/C. This reinforces the role of healthcare personnel not only as clinical providers but also as agents of social change [17].

### 4.1. Strengths and Limitations of the Study

This study highlights several strengths. First, it utilized active learning methodologies that combined theoretical instruction with practical, case‐based exercises. This approach enabled students to apply their knowledge in realistic clinical scenarios. The program was evidence‐based, developed after a thorough literature review, and integrated into the curriculum of a core nursing course, which enhanced its relevance and applicability. Furthermore, the use of a previously validated questionnaire to assess general knowledge, attitudes, and clinical management knowledge provided a standardized framework for measuring pre–post changes. The pre–post quasi‐experimental design allowed for the evaluation of changes directly related to the intervention, providing valuable insights into the potential educational value of active learning approaches in nursing education.

Several limitations should be acknowledged. Firstly, the quasi‐experimental pre–post design without a control group limits the ability to establish causal relationships between the intervention and the observed outcomes. Therefore, the findings should be interpreted as pre–post changes associated with the educational program rather than effects attributable solely to the intervention. In addition, observed changes may have been influenced by potential confounding factors, including testing effects, social desirability bias, exposure to other course content, or short‐term recall of information.

Secondly, the low internal consistency of the attitude and clinical management subscales was a limitation. This may partly be explained by the small number of items in these domains, as Cronbach’s alpha is sensitive to short scales. The heterogeneous coding structure of some items may also have limited the interpretability of the composite‐domain scores.

Thirdly, the sample was predominantly female (75.4%), which reflects the gender distribution of nursing programs but limits the possibility of sex‐based analyses. Moreover, the single‐center design and the use of a nonprobabilistic sampling strategy restrict the generalizability of the findings. Therefore, results should be interpreted with caution, as they may not be directly transferable to other educational, institutional, or cultural contexts. Future multicentre studies with more diverse samples are needed to confirm and extend these findings.

Finally, item‐level analyses were exploratory in nature and were not adjusted for multiple comparisons. Consequently, some statistically significant findings may represent false‐positive results and should be considered hypothesis‐generating rather than confirmatory. In addition, the qualitative component was exploratory and descriptive and was intended to complement the quantitative findings rather than provide an in‐depth qualitative analysis.

### 4.2. Recommendations for Future Research

Future studies should aim to use larger and more representative samples to enhance generalizability and facilitate subgroup analyses based on sex or cultural background. Additionally, incorporating longitudinal designs would enable the assessment of knowledge and skill retention over time, as well as how training translates into actual clinical practice. Future interventions could also benefit from including observational or simulation‐based assessments to assess observable clinical performance more accurately, in conjunction with self‐reported questionnaires.

Moreover, investigating the impact of complementary education on ethics, human rights, and intercultural competence may contribute to changes in attitudes and ethical decision‐making regarding FGM/C. Finally, comparing different active learning strategies, such as simulation, role‐playing, and case studies, could help identify the most effective teaching methods for nursing education on sensitive cultural practices.

### 4.3. Implications for Public Health Nursing

The findings highlight the essential role of nursing education in preparing students to identify, prevent, and manage cases of FGM/C. The integration of active learning interventions into undergraduate and continuing education programs may enhance students’ theoretical knowledge of FGM/C and support ethical reflection regarding the care of affected women and girls. Further research using reliable outcome measures and objective assessments is needed before conclusions can be drawn regarding clinical preparedness.

Strengthening nursing students’ knowledge regarding FGM/C management may contribute to improved detection, prevention, and support for women and girls at risk. Furthermore, trained nurses can serve as agents of social change by educating communities and raising awareness about the health, ethical, and legal implications of FGM/C.

Incorporating FGM/C education systematically into nursing curricula supports comprehensive public health strategies aimed at reducing the prevalence of this harmful practice and improving the quality of care for women and girls who have undergone FGM/C.

## 5. Conclusion

This one‐group pre–post educational evaluation suggests that an active learning‐based FGM/C training program was associated primarily with short‐term improvements in nursing students’ general knowledge of FGM/C. Changes in attitudes were small and should be interpreted cautiously due to the low reliability of the subscale, and no significant improvements were observed in clinical management knowledge.

Overall, the largest observed change was in general knowledge acquisition. In addition, the low internal consistency of the attitude and clinical management subscales limits the interpretability of these composite‐domain results and may have reduced the sensitivity of these measures to detect meaningful changes.

Further controlled studies using longer follow‐up periods, validated and reliable outcome measures, and objective assessments of clinical competence (e.g., simulation‐based or observed performance) are needed before firm conclusions can be drawn regarding the impact of such interventions on clinical preparedness.

Given the psychometric limitations of the attitude and clinical management subscales, conclusions regarding changes in attitudes, clinical competence, or practical preparedness should be considered preliminary and limited to the questionnaire‐measured responses.

## Funding

This research was funded by the Department of Nursing at the University of Murcia.

## Conflicts of Interest

The authors declare no conflicts of interest.

## Supporting Information

Additional supporting information can be found online in the Supporting Information section.

## Supporting information


**Supporting Information** Supporting Information S1. Detailed description of the educational intervention, including learning objectives, session structure, teaching materials, case‐based learning activities, and implementation of fidelity procedures to support reproducibility. Supporting Information S2. Detailed description of questionnaire item allocation, coding procedures, response structures, score interpretation, and dimensional classification used for quantitative analysis.

## Data Availability

The study protocol, intervention materials, and questionnaire scoring system are publicly available on the Open Science Framework (OSF): https://osf.io/agj5q. Anonymized individual‐level datasets are not publicly available due to ethical and confidentiality considerations related to the use of a student population within a university setting. However, deidentified data may be requested from the corresponding author upon reasonable request and subject to institutional ethical approval.
