# Supplementary material for: Effects of Training Nursing Students on Female Genital Mutilation: A Quasi‐Experimental Study With Pre‐ and Postintervention Assessments
Source: Nurs Res Pract. 2026 Oct 11;2026:9450657. doi: 10.1155/nrp/9450657 (PMC13655381; doi:10.1155/nrp/9450657)
Supplement: Supplementary file 1 [file NRP-2026-9450657-s001.docx]

**Supplementary Material**

**Supplementary Material S1. Detailed description of the educational intervention**

This document provides a comprehensive description of the active learning-based training programme on female genital mutilation/cutting (FGM/C) implemented in this study. It is provided to ensure transparency, reproducibility, and methodological rigour. The material includes the learning objectives, session structure, teaching resources, case vignettes, and intervention fidelity procedures.

**1. Overview of the intervention**

The educational intervention was designed to improve nursing students’ knowledge, attitudes, and clinical management skills regarding Female Genital Mutilation/Cutting (FGM/C). The programme was based on active learning principles and problem-based learning (PBL), fostering critical thinking, clinical reasoning, and the application of theoretical knowledge to clinical practice.

The intervention was implemented during the 2024–2025 academic year as part of the course “Nursing and Women’s Health”.

**2. Learning objectives**

At the end of the programme, students were expected to:

- Define Female Genital Mutilation/Cutting (FGM/C) and describe its WHO classification.
- Identify cultural, social, and health determinants associated with FGM/C.
- Recognise short- and long-term physical, psychological, and obstetric consequences.
- Understand the international, national, and regional legal frameworks related to FGM/C.
- Apply clinical protocols for prevention, detection, and management of FGM/C cases.
- Develop appropriate communication strategies with affected women, girls, and families.
- Reflect critically on cultural practices from a human rights-based and ethical perspective.

**3. Structure and delivery of the programme**

The programme consisted of two structured components:

**3.1 Theoretical session**

- Total participants: 69 nursing students
- Duration: 2 hours
- Format: Plenary lecture
- Teaching resource: PowerPoint presentation

Content included:

- Definition and WHO classification of FGM/C
- Epidemiology and global distribution
- Cultural, religious, and social factors
- Health consequences (short- and long-term)
- Human rights implications
- Legal and ethical framework
- Clinical identification and risk assessment

**3.2 Practical session**

- Total participants: 69 students
- Organisation: 3 small groups of 23 students each
- Duration: 2 hours per group
- Method: case-based learning in small groups

Each group worked on structured clinical scenarios guided by the teaching staff.

Activities included:

- Analysis of clinical cases
- Application of the regional protocol
- Group discussion and decision-making exercises
- Development of nursing care plans
- Communication strategies in culturally sensitive contexts

**4. Teaching methodology**

The intervention was based on active learning strategies, specifically:

- Problem-Based Learning (PBL)
- Case-Based Learning (CBL)
- Collaborative group discussion
- Clinical reasoning exercises

These methodologies promoted student engagement and integration of theoretical and practical knowledge.

**5. Clinical case vignettes used in training**

**Case 1: Clinical care of a woman with FGM/C**

A woman with a history of female genital mutilation/cutting (FGM/C) attends a healthcare consultation requiring clinical assessment and care planning. Students were expected to identify appropriate nursing actions related to comprehensive care, complication prevention, and multidisciplinary management.

**Case 2: Culturally sensitive clinical interview**

A patient from a country with a high prevalence of FGM/C attends a healthcare consultation. Students were required to consider culturally appropriate communication strategies and conduct a respectful and adapted clinical interview, ensuring trust-building and appropriate information gathering.

**Case 3: Safeguarding minor at risk of FGM/C**

A minor presenting identifiable risk factors for FGM/C undergoes a routine clinical examination, with no evidence of genital cutting detected. Students were expected to determine appropriate preventive, legal, and safeguarding nursing actions in accordance with clinical and ethical guidelines.

**Case 4: Pregnancy and history of FGM/C**

A pregnant woman discloses a history of FGM/C performed during childhood. Students were required to consider obstetric implications, potential complications, and appropriate clinical management, including referral pathways and culturally sensitive care.

**6. Facilitators and implementation**

- Facilitators: same teaching team throughout the intervention
- Expertise: nurses and university lecturers with teaching experience in women’s health and previous training in FGM/C prevention and management.
- Facilitation method: each case discussion followed a structured sequence consisting of individual case review, small-group discussion, identification of key clinical and ethical issues, and facilitator-led debriefing.
- Consistency: all sessions followed the same structured teaching script

**7. Intervention fidelity**

To ensure standardization of delivery:

- A predefined educational protocol was used for all sessions
- Content and timing were consistent across groups
- No deviations from the planned intervention were recorded
- Attendance was monitored for all sessions

**8. Educational materials**

The following materials were used:

- PowerPoint presentation (theoretical session)
- Regional protocol: Protocol for the Prevention and Health Action against Female Genital Mutilation in the Region of Murcia
- Structured clinical case vignettes
- Group discussion guidelines

**9. Theoretical framework**

- The intervention was grounded in:
- Active Learning Theory
- Problem-Based Learning (PBL)
- Constructivist learning approach

These frameworks support student-centred learning and the development of clinical reasoning and decision-making skills.

**Supplementary Material S2. Questionnaire Scoring System**

For most knowledge items, higher values reflected greater knowledge. For attitude items, score interpretation depended on the analytical coding; lower scores indicated more appropriate attitudes toward FGM/C (greater rejection of the practice and its medicalization). Clinical management items (P11–P14) retained their original scoring structure based on the pattern of management actions selected.

To facilitate transparency and reproducibility, the table below provides information on domain allocation, original response structure, analytical recoding, response format, score direction, and interpretation of each questionnaire item.

| **Item** | **Domain** | **Original Response Structure** | **Analytical Recoding** | **Response Format** | **Direction of score** | **Score Interpretation** |
| --- | --- | --- | --- | --- | --- | --- |
| 1. Do you know about female genital mutilation (FGM)? | General knowledge | No / Yes / Do not know | 0=No, 1=Do not know/No response, 2=Yes | Ordinal categorical item | Higher scores indicate greater knowledge. | Higher scores reflected greater awareness of FGM/C. |
| 2. If you answered YES to the previous question, do you know how many types there are? | General knowledge | Single-choice item | Recoded according to correctness of selected option | Recorded ordinal item |  | Higher scores reflected more accurate identification of FGM/C typologies. |
| 3. Could an assessment determine if female genital mutilation (FGM) has occurred? | General knowledge | No / Yes / Do not know | 0=No, 1=Do not know/No response, 2=Yes | Ordinal categorical item |  | Higher scores reflected greater perceived ability to identify FGM/C. |
| 4. What do you think are the reasons why it is done? | General knowledge | Multiple-response item | Recoded according to number of guideline-consistent responses selected | Recorded ordinal item |  | Higher scores reflected broader recognition of sociocultural motivations associated with FGM/C. |
| 5. Do you think the practice of FGM should be respected for geographical, cultural, religious, or other reasons? | Attitudes | No / Yes / Do not know | 0=No, 1=Do not know/No response, 2=Yes | Ordinal categorical item | Lower scores indicate greater rejection of FGM/C. | Lower scores reflected greater disagreement with respecting FGM/C for cultural, religious, geographical, or other reasons. |
| 6. If a health check were conducted, would you participate in FGM as a professional? | Attitudes | No / Yes / Do not know | 0=No, 1=Do not know/No response, 2=Yes | Ordinal categorical item | Lower scores indicate lower acceptance of FGM/C medicalization. | Lower scores reflected lower willingness to participate in the medicalization of FGM/C. |
| 7. Have you encountered any cases in your practice, or do you know of any similar cases? | General Knowledge | Yes / No / Do not know | 0=No, 1=Do not know/No response, 2=Yes | Ordinal categorical item | Lower scores indicate more appropriate attitudes toward FGM/C. | Higher scores reflected greater awareness of previous exposure to FGM/C cases. |
| 8. What health problems are associated with Female Genital Mutilation (FGM)? | General Knowledge | Multiple-response item | Recoded according to number of correctly identified health consequences | Ordinal categorical item | Higher scores indicate greater knowledge. | Higher scores reflected broader recognition of FGM/C-related health consequences. |
| 9. Is there any legislation regarding female genital mutilation (FGM) in our country? | General knowledge | No / Yes / Do not know | 0=No, 1=Do not know/No response, 2=Yes | Ordinal categorical item |  | Higher scores reflected greater awareness of legislation related to FGM/C. |
| 10. Do you know of any protocols or guidelines for action? | General knowledge | No / Yes / Do not know | 0=No, 1=Do not know/No response, 2=Yes | Ordinal categorical item |  | Higher scores reflected greater awareness of protocols and guidelines related to FGM/C. |
| 11. What is the recommended course of action for a woman with FGM? | Clinical management knowledge | Multiple-response clinical scenario | Original scoring retained | Recorded ordinal item | Higher scores indicate better clinical management knowledge. | Higher scores reflected broader identification of recommended healthcare management actions. |
| 12. What steps would you take to conduct an adequate clinical interview with a patient from a country where female genital mutilation is practiced? | Clinical management knowledge | Multiple-response clinical scenario | Original scoring retained | Recorded ordinal item |  | Higher scores reflected broader identification of recommended safeguarding and clinical actions. |
| 13. What actions would you take if you encountered a minor girl at risk for FGM who undergoes a genital examination and is found not to have experienced genital mutilation? | Clinical management knowledge | Multiple-response clinical scenario | Original scoring retained | Recorded ordinal item |  | Higher scores reflected broader identification of preventive and protection-related actions. |
| 14. How would you respond to a pregnant woman who tells you she underwent FGM as a child? | Clinical management knowledge | Multiple-response clinical scenario | Original scoring retained | Recorded ordinal item |  | Higher scores reflected broader identification of obstetric and multidisciplinary management actions. |
| 15. Have you attended any courses or talks on female genital mutilation (FGM)? | Descriptive item (excluded from dimensional scores) | No / Yes | 0=No, 1=Yes | Dichotomous item | Higher scores indicate previous participation in FGM/C-related training. | Item assessed previous participation in FGM/C-related educational activities. |

**Note.** P15 was excluded from dimensional and total questionnaire scores and analyzed separately as a descriptive variable. Clinical management items (11–14) retained the original scoring structure of the validated instrument due to their complex clinical-response format. Low internal consistency observed in the attitude and clinical management of subscales should be considered when interpreting composite-domain findings.
